# Radiation-induced prodrug activation: extending combined modality therapy for some solid tumours

**DOI:** 10.1038/s41416-022-01746-1

**Published:** 2022-02-25

**Authors:** Nicola J. Farrer, Geoff S. Higgins, Ian H. Kunkler

**Affiliations:** 1 Chemistry Research Laboratory, University of Oxford, 12 Mansfield Road, Oxford, OX1 3TA UK; 2 Department of Oncology, MRC Oxford Institute for Radiation Oncology, University of Oxford, Oxford, UK; 3 Cancer Research UK Edinburgh Centre, Institute of Genetics and Cancer, University of Edinburgh, Edinburgh, EH4 2XU UK

**Keywords:** Radiotherapy, Drug development

## Abstract

Combined chemoradiotherapy is the standard of care for locally advanced solid tumours. However, systemic toxicity may limit the delivery of planned chemotherapy. New approaches such as radiation-induced prodrug activation might diminish systemic toxicity, while retaining anticancer benefit. Organic azides have recently been shown to be reduced and activated under hypoxic conditions with clinically relevant doses of radiotherapy, uncaging pazopanib and doxorubicin in preclinical models with similar efficacy as the drug, but lower systemic toxicity. This approach may be relevant to the chemoradiation of glioblastoma and other solid tumours and offers potential for switching on drug delivery from implanted devices. The inclusion of reporters to confirm drug activation, avoidance of off-target effects and synchronisation of irradiation with optimal intratumoral drug concentration will be critical. Further preclinical validation studies of this approach should be encouraged.

## Introduction

Concurrent delivery of chemoradiotherapy (CRT) is the standard of care for many locally advanced solid tumours, including head and neck, lung, urological, gastrointestinal, cervix, sarcomas and brain [1]. The rationale for CRT draws on the Steel paradigm of spatial cooperation and radiation sensitisation [2]. Some chemotherapeutics (e.g. cisplatin) sensitise cancer cells to radiotherapy (RT), exacerbating DNA damage and inhibiting repair [3]. However, untargeted chemotherapeutics can cause appreciable systemic toxicity [4]. New approaches to CRT such as radiation-induced prodrug activation might reduce systemic toxicity while retaining anticancer benefit, with the potential for an improved therapeutic strategy.

## Promising prodrugs

Under conditions of chronic hypoxia, organic azides can be enzymatically reduced to amines, and this has been exploited to release a fluorophore, for imaging. Compared with normoxic conditions, increased fluorescence was initially observed at 3% O_2_ and progressively increased with lower oxygen concentrations (1%, 0.5% and <0.1% O_2_) [5]. It is unclear whether the activation of this compound would differ under conditions of acute or cycling hypoxia. Recently, organic azides have also been shown to be reduced and activated in preclinical models under hypoxic conditions, using clinically relevant doses of external beam RT. This has been exemplified by the uncaging of anticancer agents pazopanib and doxorubicin, confirmed by chromatography [6]. The efficiency of the azide reduction by RT—postulated to be mediated by free radicals—is highly dependent on the precise chemical structure, with fluorination of the azido caging group markedly improving RT conversion and release of the active agent. Good prodrug stability in whole blood was observed in the absence of RT, and in vivo work demonstrated prolonged survival and reduced systemic toxicity compared to the free drug (as determined by blood markers and histology). The potential for the enzymatic activation of these compounds may add a further layer of complexity, including potential off-target effects in tissues with physiological hypoxia (liver, retina and possibly muscle and bone). However, the physiological hypoxia observed in these tissues is typically much less extreme than those seen in solid tumours—potentially enabling a window of hypoxia by which the prodrugs are activated by RT, but not under normal conditions.

Overall, the possibility for using an external X-ray beam to uncage functional groups (amine, alcohol) which are common in existing anticancer agents, in a hypoxic environment, provides a new paradigm of spatial and temporal control over the treatment of solid tumours. Although the reported organic azido compounds were not specifically targeted to tumours, RT offers a significant degree of targeted activation. If prodrug activation can be highly controlled with synchronised localised CRT, tumour cell kill can be maximised while minimising systemic toxicity.

The potential for RT activation of metal complexes (particularly cobalt) [7, 8] and metalloporphyrins [9] for application in cancer treatment is well-documented. Furthermore, an X-ray analogue of photodynamic therapy (PDT) is under development—termed PDT-X [10]. This is similar in mechanism to PDT, in that it uses a sensitiser or a nanoparticle, or both [11]. PDT-X is partly mediated by singlet oxygen, in addition to other radicals and reactive oxygen species. Therapeutic activity under different oxygen concentrations depends on the chosen sensitiser, and small clinical trials have shown promise [10], and the use of PLGA nanoparticles has been validated for PDT-X of verteporfin [12].

Although the dominant mechanisms of action for PDT (and by likely analogy, PDT-X) may depend on individual cancer [13], PDT-X is likely to be less effective for severely hypoxic tumours. Notably, there is preclinical evidence that PDT can stimulate the host immune system against tumour cells including activating anti-tumour T lymphocytes; [14] it remains to be seen if the same is true for PDT-X. Tumour hypoxia remains a major cause of resistance to RT and chemotherapy, and clinical studies of hypoxia-activated prodrugs have been disappointing, in part due to the inclusion of patients with insufficiently hypoxic tumours [15]. Therefore, the development of relatively simple organic azide prodrugs which can be activated by RT, under hypoxia, is encouraging.

## Suitability of organic azide approach for different cancers treated by CRT

**Fig. 1:**
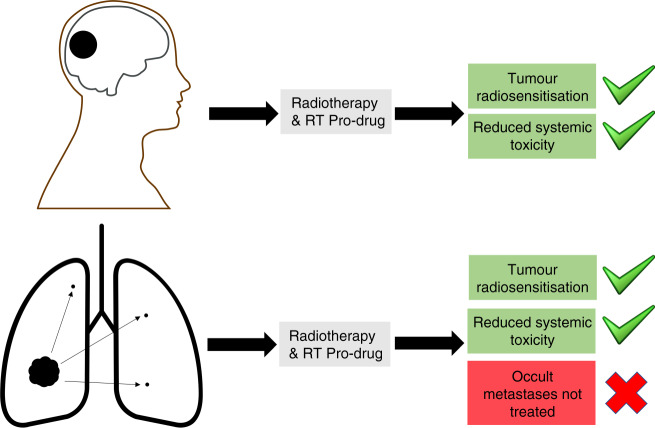
The potential benefit of radiotherapy-activated organic azide prodrugs by the clinical scenario. Greatest benefit is perceived where chemotherapy is predominantly added to radiotherapy treatment (e.g. glioblastoma) to radiosensitise the localised tumour (top). Where chemotherapy plays an important role in treating occult metastatic disease (e.g. locally advanced small cell lung cancer, bottom), this will be less well-addressed.

The principal application of the RT-activated organic azide approach is likely to be settings where delivery of optimal dose and duration of conventional CRT is limited by systemic toxicity (see Fig. 1). It is possible that RT could be used to switch on drug delivery from implanted devices [6], for example, we suggest at the margins of resection of glioblastoma to treat residual disease. It may also be relevant for the treatment of head and neck cancer (HNSCC), in which chemotherapy is used primarily for radiosensitisation, with a more modest effect on reducing metastases [16].

A limitation of the RT-activated organic azide prodrug approach is the treatment of micrometastatic spread. There might be a role for achieving cytoreduction in the primary tumour, where systemic toxicity prevents further chemotherapy, and the potential for therapeutic gain and reduced radiotoxicity from RT-induced prodrug activation at therapeutic or sub-therapeutic doses. Specific development of prodrugs of potent DNA damage repair (DDR) inhibitors such as platinum compounds, DNA-dependent protein kinase (DNA-PK) and poly-ADP ribose polymerase (PARP) inhibitors, where the current treatment is RT alone (e.g. stereotactic ablative body radiotherapy (SABR) for NSCLC and oligometastases) is likely to be highly beneficial. This might be to either increase tumour control (same RT dose), or to reduce RT dose without compromising tumour control. This may also potentially increase the immunostimulatory effects of RT [17], since the use of platinum [18] or PARP prodrugs is likely to increase the amount of DNA damage, micronuclei formation and cyclic GMP-AMP synthase (cGas) activation [19], which may subsequently increase the immunotherapy response.

## Dose and dose rate

Previous attempts to develop orally available prodrugs (such as Satraplatin, which reached Phase III clinical trials) has demonstrated the potential for non-linear and highly variable absorption pharmacokinetics [20], which would further complicate attempts to externally activate prodrugs. Intravenous administration is therefore the most likely administration route for these prodrugs. Since timing of RT application following administration of the prodrug is an important consideration, IV delivery might also offer tighter control, which ideally would be optimised for an individual patient, by low-cost imaging of prodrug accumulation and excretion. There are potential benefits in waiting for vascular clearance of the prodrug which has not accumulated in the tumour. However, specification of a time delay between administration of prodrug and activation (particularly which might be patient-specific) can add considerable complexity in a typical clinical setting. Administration of the RT as soon as possible after administration for prostate cancer (as per palladium-based porphyrin PDT Phase III clinical trials [21]) can cause vascular shut down of a tumour, and can be highly effective. This approach was also taken in ultrasound clinical trials for liver cancer (e.g. NCT02181075) [22]. However, for some modes of activation and cancer locations, such as the early glioblastoma BNCT clinical trials in the 1950’s, a similar approach resulted in unacceptable off-target damage [23].

The recently reported doxorubicin azido prodrug demonstrated a dose-dependent response [6], with 60 Gy achieving a 50% prodrug conversion to doxorubicin in vitro, and 6 Gy inhibiting tumour growth in tumour-bearing mice. Chemical modification may further improve prodrug activation without compromising stability, and dose rate modulation (e.g. FLASH) can also be explored. However, if doses of >6 Gy are required in a single RT session for sufficient prodrug activation, this would limit use to SABR-type scenarios. If prodrug activation can be achieved with 2 Gy, this broadens applicability to conventional fractionation.

A typical CRT course might consist of RT of 2 Gy daily (M-F), over a 6-week period, with chemotherapy administered on 3 days during this period. Maximising the benefits of the RT activation approach will have to address the substantial clinical complexity of scheduling prodrug delivery before each RT session with pulses of intravenous chemotherapy. This raises the possibility of either reducing frequency of the RT sessions (e.g. 1 session per week for 6 weeks, giving time for the chemotherapy to take effect), or daily RT sessions but with a shorter treatment cycle.

## Conclusions

The development of RT-activated organic azide prodrugs could be an important addition to the armamentarium of CRT for some solid tumours, irrespective of hypoxic status. Such RT-activated prodrugs might reduce local and systemic toxicity. Inclusion of drug activation reporters, and synchronisation of irradiation with optimal intratumoural drug concentration will be important. We encourage further preclinical validation studies of this approach.
